# *Streptococcus anginosus*: A new pathogen of superficial gastritis, atrophic gastritis, and gastric cancer

**DOI:** 10.17305/bb.2024.10705

**Published:** 2024-10-01

**Authors:** Fengting Guo, Lanfang Li, Lifang Li

**Affiliations:** 1 Guangxi University of Chinese Medicine, Nanning, Guangxi, China; 2 Institute of Pharmacy and Pharmacology, University of South China, Hengyang, Hunan, China; 3 Department of Traditional Chinese Medicine, Shenzhen Second People’s Hospital, Shenzhen, Guangdong, China

**Keywords:** Atrophic gastritis (AG), gastric cancer (GC), *Helicobacter pylori*, *Streptococcus anginosus*, superficial gastritis (SG)

## Abstract

A wealth of research indicates that superficial gastritis (SG) and atrophic gastritis (AG) are precursors to gastric cancer (GC). While *Helicobacter pylori* has long been recognized as a key player in GC development, recent findings by Fu et al. have identified *Streptococcus anginosus* as an emerging pathogen that can trigger SG, AG, and GC. *S. anginosus*, a gram-positive coccus, leverages its surface protein *T. pallidum* membrane protein C (TMPC) to engage with the annexin A2 (*ANXA2*) receptor of gastric epithelial cells, facilitating its colonization and invasion in the gastric mucosa. This leads to an upregulation of proinflammatory chemokines Ccl20 and Ccl8, causing prolonged effects on gastric barrier function and microbiota homeostasis, leading to SG. Moreover, these bacteria activate the mitogen-activated protein kinase (MAPK) signaling pathway, which is associated with the development of AG and GC. Importantly, inhibiting TMPC or knocking down *ANXA2* can reduce *S. anginosus* colonization and invasion, lowering the chances of SG, AG, and GC. This paper highlights the molecular mechanisms of *S. anginosus* in SG, AG, and GC, emphasizing the importance of a multi-pathogen strategy in gastric disease management and the need for further investigation into the role of *S. anginosus* in GC progression.

Superficial gastritis (SG) is a prevalent digestive disorder, characterized by the hallmark infiltration of inflammatory cells within the gastric mucosa. This condition is often accompanied by symptoms, such as abdominal pain, belching, and a reduced appetite. If left untreated, SG may evolve into atrophic gastritis (AG), which is primarily characterized by injury to gastric mucosal epithelial cells, atrophy or disappearance of glands, decreased gastric secretion function, and potential development of intestinal metaplasia (IM) and dysplasia [1, 2]. IM and dysplasia are recognized as precancerous gastric conditions [3, 4]. Consequently, both SG and AG are linked to an increased risk of gastric cancer (GC).

GC is characterized as a primary malignant tumor that arises in the stomach. The development and progression of GC are the result of a chronic process, influenced by a multitude of factors [5]. Among them, *Helicobacter pylori* infection is recognized as an important factor [6]. *H. pylori* is a microaerobic gram-negative bacterium with flagella that plays a crucial role in gastrointestinal mucosa colonization. *H. pylori* colonizes nearly half of the global population’s gastrointestinal mucosa, with varying prevalence rates across different regions [7]. Epidemiological studies have indicated that *H. pylori* infection is a substantial risk factor for the evolution of SG, AG, and GC [6]. Consequently, therapeutic intervention targeting has become a critical strategy for preventing SG, AG, and GC.

Recent studies have revealed the presence of a complex microbial environment within the stomach. While *H. pylori* is a widely recognized pathogen [8], other non-*H. pylori* pathogens, such as *Streptococcus anginosus*, a gram-positive coccus, are also prevalent in the oral cavity, nasopharynx, gastrointestinal tract, and vagina, and have been linked to suppurative infections. Owing to its acid tolerance, with a pH range of 3–5, *S. anginosus* can adapt to the acidic environment in the stomach [9, 10]. Fu et al. [11] reported that *S. anginosus* not only colonizes the stomach but also causes the development of SG, AG, and GC.

Fu et al. developed an infection mouse model by oral administration of *S. anginosus* to investigate its pathogenic role in the stomach. They found that short-term infection of mice with *S. anginosus* induces SG. Within two weeks after *S. anginosus* infection, *S. anginosus* colonizes the basal part of the gastric mucosa, and the expression of pro-inflammatory chemokines *Ccl20* and *Ccl8* is upregulated, triggering an acute inflammatory response. Three months after infection with *S. anginosus*, neutrophils persistently infiltrate the gastric tissue and aggregate to form lymphocyte foci within the gastric submucosa, consequently leading to the development of chronic gastritis in mice. In addition, gastroscopic biopsy analysis has shown a higher relative risk of AG, IM, and GC in patients co-infected with *S. anginosus* and *H. pylori* compared to those with *H. pylori* infection alone. Together, these findings indicate that short-term *S. anginosus* infection can induce SG and suggest a potential synergistic effect between *S. anginosus* and *H. pylori* in the exacerbation of gastric mucosal inflammation.

Moreover, long-term infection with *S. anginosus* can lead to progressive gastric lesions characterized by AG, IM, and dysplasia. Fu et al. found that mice infected with *S. anginosus* exhibit mild cellular atrophy nine months after infection and moderate to severe cellular atrophy 12 months after infection. IM and dysplasia also develop in mice infected with *S. anginosus* by 12 months, as demonstrated by tissue section images, H&E staining, and Alcian blue staining. Additionally, germ-free mice were successfully colonized by *S. anginosus* in the gastric mucosa, which led to the development of IM without the influence of other gastric microbes. These findings collectively demonstrate that long-term infection with *S. anginosus* directly causes gastric precancerous lesions, including AG, IM, and dysplasia.

To elucidate the effects of *S. anginosus* on GC, Fu et al. established two distinct mouse GC models. In the N-methyl-N-nitrosourea (MNU)-induced GC mouse model, mice infected with *S. anginosus* have larger and more numerous tumors than control mice treated with MNU only. It was inferred that *S. anginosus* increases the incidence of GC in mice. In the YTN16 GC xenograft model, tumor volume and weight are significantly increased in mice injected with *S. anginosus* compared with the control group. It was deduced that *S. anginosus* accelerates the growth of gastric tumors. Collectively, these results identified that *S. anginosus* accelerates the formation and progression of GC.

*S. anginosus* can not only directly cause SG, AG, and GC but also promote the development of these conditions by altering the gastric environment. Claudin18 (CLDN18) is a tight junction protein specifically expressed in the stomach. After long-term infection with *S. anginosus*, there is a significant loss of CLDN18 throughout the gastric body or gland. This suggests that *S. anginosus* impairs gastric barrier function, the destruction of which is an important marker of GC progression. To elucidate changes in the gastric microbiota following *S. anginosus* infection, Fu et al. conducted 16s rRNA sequencing on gastric mucosa samples from infected mice. Compared to healthy subjects and those with SG, patients with precancerous lesions and GC show an increase in pathogenic oral commensal bacteria, such as *Prevotella* and *Aggregobacter*, and have decreased numbers of probiotics, such as *Bifidobacterium pseudolongum* and *Lactobacillus*. These findings indicate that *S. anginosus* can disrupt the gastric barrier function and disturb the homeostasis of gastric microbiota, thereby creating a gastric environment conducive to the formation and progression of SG, AG, and GC.

Finally, Fu et al. investigated the molecular mechanisms of *S. anginosus*. They found that *T. pallidum* membrane protein C (TMPC), a surface protein of *S. anginosus*, interacts directly with annexin A2 (*ANXA2*), which serves as a co-receptor of TMPC. This interaction enables *S. anginosus* to colonize the gastric mucosa. Short-term infection with *S. anginosus* can upregulate the expression of proinflammatory chemokines Ccl20 and Ccl8, triggering SG. Long-term infection activates the mitogen-activated protein kinase (MAPK) signaling pathway, specifically the ERK, JNK, and p38 subfamilies, which contribute to the progression of AG and GC. Inhibiting TMPC or knocking down *ANXA2* reduces *S*. *anginosus* colonization and invasion, lowering the occurrence of SG, AG, and GC (Figure 1). However, the role of *S. anginosus* in gastric disease is complex, and further research is needed to understand its specific mechanism and potential pathways it may provide for GC prevention and treatment.

*S. anginosus* emerges as a promising biomarker for the early diagnosis of GC. Zhou et al. [12] revealed the presence of *S. anginosus* in the feces of patients with gastric precancerous lesions, as well as in those with both early and advanced GC. Notably, *S. anginosus* is detected most frequently in the feces of early GC patients, with a detection rate exceeding that in both chronic gastritis and advanced GC cohorts. Comparative analysis of fecal samples from patients with colorectal adenoma, colorectal cancer, esophageal cancer, or inflammatory bowel disease further accentuates the specificity of *S. anginosus* to GC, underscoring its potential as a non-invasive and facile diagnostic tool. In the future, the detection technology of *S. anginosus* is expected to be further optimized and standardized to ensure its effectiveness as an early screening tool for GC. The high specificity of *S. anginosus* in stool samples from GC patients, combined with its role in promoting GC progression, underscores the need for an in-depth study of the complex relationship between *S. anginosus* and GC.

The higher prevalence of *H. pylori* in the Chinese population, compared to Europeans and Americans, is attributed to dietary differences. The preference of Westerners for separate meal systems reduces the risk of *H. pylori* infection. Muzaheed [13] found that *S. anginosus* infection is associated with oral hygiene, with dental plaque identified as the main reservoir of *S. anginosus*. *S. anginosus* can colonize the throat, nasopharynx, gastrointestinal tract, and urogenital tract through oral transmission [14]. Adopting separate meals, maintaining oral cleanliness, disinfecting utensils, and implementing other measures to sever transmission routes can impede the spread of both *S. anginosus and H. pylori*, thereby effectively preventing the development of SG, AG, and GC. The study by Fu et al. not only points to the role of *S. anginosus* in promoting these gastric disorders but also suggests the presence of other microorganisms associated with SG, AG, and GC in the stomach. This highlights the importance of targeted public health interventions focusing on diet and oral health, particularly in regions with a high incidence of GC, such as China.

*S. anginosus* is a newly discovered pathogen associated with SG, AG, and GC. This indicates that anti-*H. pylori* treatment alone may not be sufficient to completely prevent the occurrence of SG, AG, and GC. It is expected that targeted prevention and control of *S. anginosus* infection will enhance the effectiveness of SG, AG, and GC prevention. Current guidelines recommend standard triple therapy as the first-line treatment for *H. pylori* infection, consisting of a proton pump inhibitor and two antibiotics (clarithromycin and amoxicillin/metronidazole) [15]. It is important to note that *H. pylori* is a gram-negative organism, while *S. anginosus* is gram-positive. This difference in biological characteristics suggests that anti-*H. pylori* drugs may not effectively treat *S. anginosus* infection. In conclusion, the findings of Fu et al. emphasize the importance of addressing multiple pathogens in the management of gastric diseases. Exploring new treatment options may lead to improved optimization of SG, AG, and GC treatment regimens in the future.

**Figure 1.**
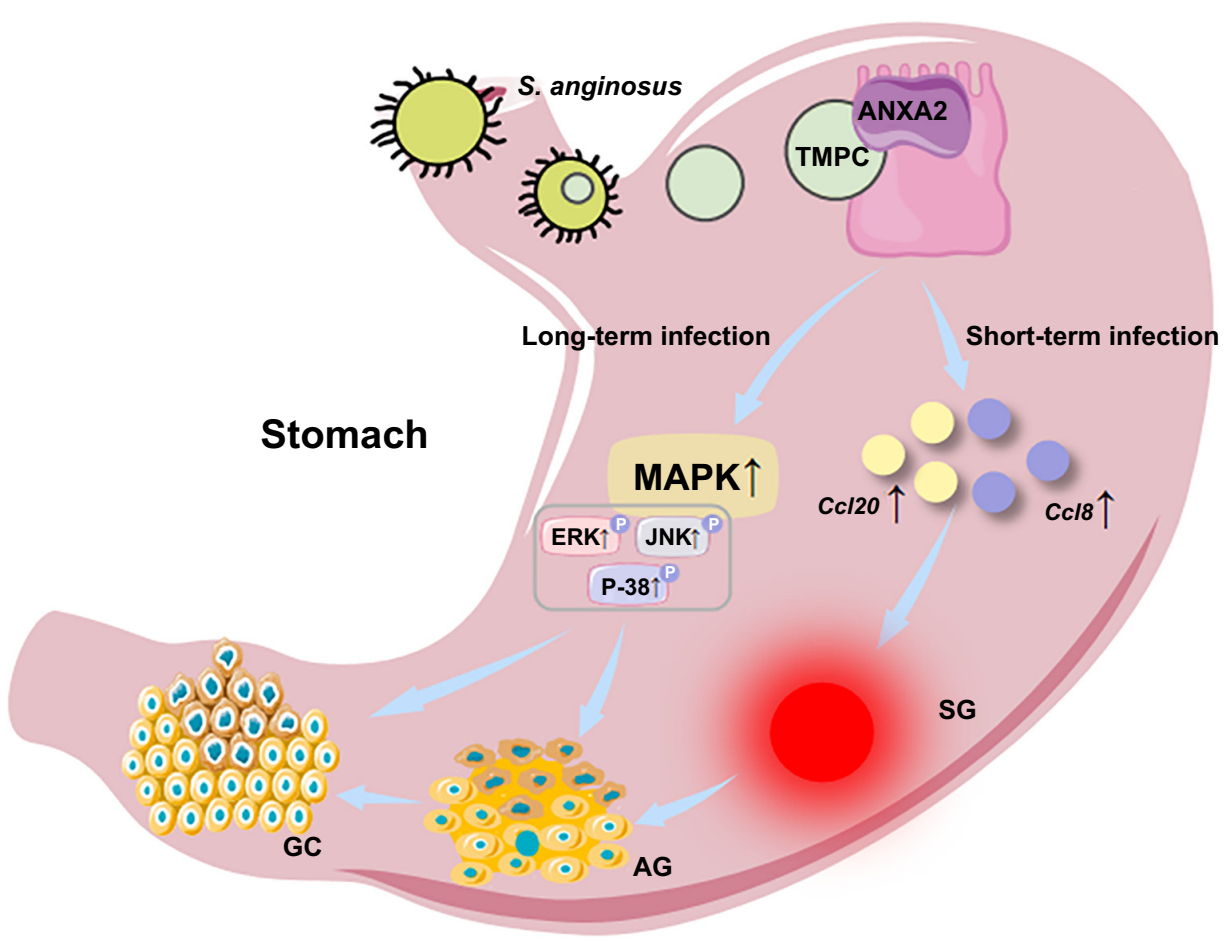
**Specific molecular mechanisms of SG, AG, and GC caused by *S. anginosus* infection.** The surface protein TMPC of *S. anginosus* directly interacts with *ANXA2* in gastric epithelial cells, enabling it to colonize and invade the gastric mucosa. Short-term infection with *S. anginosus* can upregulate the expression of proinflammatory chemokines Ccl20 and Ccl8, trigger SG. Long-term infection with *S. anginosus* promotes MAPK signaling and activates the ERK, JNK, and p38 subfamilies, which are involved in the progression of AG and GC. Inhibition of TMPC or knockdown of *ANXA2* reduces *S. anginosus* colonization and invasion, thereby reducing the incidence of SG, AG, and GC. SG: Superficial gastritis; AG: Atrophic gastritis; GC: Gastric cancer; TMPC: *T. pallidum* membrane protein C; MAPK: Mitogen-activated protein kinase.
